# Non-Suicidal Self-Injury in Eating and Feeding Disorder Patients: Characteristics and Clinical Implications in a Group of Referred Female Adolescents

**DOI:** 10.3390/children11080947

**Published:** 2024-08-06

**Authors:** Gianluca Sesso, Cristina Mazzullo, Elena Valente, Francesca Ditaranto, Pamela Fantozzi, Vittorio Belmonti, Stefano Berloffa, Francesca Placini, Raffaella Tancredi, Gabriele Masi, Annarita Milone

**Affiliations:** 1 Social and Affective Neuroscience Group, Molecular Mind Lab, IMT School for Advanced Studies, Piazza San Francesco, 55100 Lucca, Italy; 2 Department of Child and Adolescent Psychiatry and Psychopharmacology, IRCCS Stella Maris Foundation, Viale del Tirreno 331, 56128 Pisa, Italy; 3 Clinica di Neuropsichiatria dell’Infanzia e dell’Adolescenza, Ospedale Pediatrico-Microcitemico, Via Jenner s.n.c., 09121 Cagliari, Italy

**Keywords:** non-suicidal self-injury, feeding and eating disorders, anorexia nervosa, bulimia nervosa, emotional dysregulation

## Abstract

Background: Non-suicidal self-injury (NSSI) and Feeding or Eating Disorders (FEDs) often coexist during adolescence with reciprocal influences on their clinical picture. The present study aimed to identify differences and similarities in the clinical presentation of young patients with both conditions compared to those with the two non-comorbid disorders. Methods: We consecutively recruited forty-five female patients aged between 11 and 18 at our third-level hospital and subdivided them into three groups (NSSI: n = 15; FED: n = 15; NSSI + FED: n = 15). Patients underwent a full clinical assessment. Results: Based on our results, the NSSI + FED group was characterized by higher rates of binging/purging behaviors, greater prevalence of Cyclothymic Disorder, and a more severe clinical presentation compared to the non-comorbid groups. Moreover, higher levels of suicidal ideation were found in the NSSI + FED group. Pharmacological treatment patterns also differed, with SSRI being prescribed more frequently to NSSI + FED patients while mood stabilizers were prescribed more frequently to NSSI ones. A Principal Component Analysis identified four main dimensions: “Body Image” impairment was more pronounced in NSSI + FED patients, indicating negative attitudes towards their own body; “Metacognition” deficits were higher in NSSI than FED. Conclusions: The present study underscores distinctive clinical features in patients with comorbid NSSI and FED, emphasizing the urgent need for tailored intervention strategies focusing on specific symptom domains.

## 1. Introduction

Non-Suicidal Self-Injury (NSSI) is a directed intentional behavior without explicit suicidal intent, aimed to partially damage body surface, that induces bleeding, bruising, or suffering (e.g., through cutting, burning, stubbing, hitting, or scrubbing) [1]. NSSI has been recently proposed in the research section of the last edition of the Diagnostic and Statistical Manual of Mental Disorders (DSM-5-TR) [2] as a condition in which self-injurious behavior is present for five or more days in the last twelve months. In the last decades, due to the massive increase in its incidence rate, NSSI has become a serious mental health problem that involves young people from all over the world [3]. Its prevalence in community samples of youth is greatly increased in the last few years, being estimated by previous studies at ~23% for lifelong rates and ~19% in the preceding year [4]. Onset is usually between 11 and 15 years with a peak around 15–17 years, and remission during late adolescence or young adulthood [5,6], although rates may vary significantly during adolescence [7]. Female gender has been identified as a major risk factor for NSSI; indeed, a recent meta-analysis reported that female adolescents and adults were more likely to engage in NSSI than males [8]. The reason for the prevalence of NSSI is not known and as with all complex behaviors, there are likely multiple factors at play. Based on what emerges from the literature, it is possible that this higher prevalence is the result of a mixture of biological (especially hormonal) and environmental factors (different functions of NSSI) [8].

Great relevance is posed to the association between NSSI and suicidal attempts [6,8,9] which is also accounted for by higher comorbidity rates with anxiety, depressive, and personality disorders [10].

Feeding and eating disorders (FEDs) are extremely invalidating conditions with possible lethal outcomes and great social burden which often significantly affect physical health and psychosocial functioning. Prevalence rates significantly increased in the last fifty years likely due to multiple factors including changes in eating habits [11]. In the DSM-5-TR, FEDs have been integrated within a single category and diagnostic criteria have changed for both Anorexia Nervosa (AN) and Bulimia Nervosa (BN) [2]. Prevalence studies indicate that children may develop AN from the age of eight onwards, while BN appears to be rarer below the age of 14 [12]. Mortality rates have been estimated around ~0.5%, although only one patient in three receives adequate clinical care and is effectively treated. One third of patients display long-lasting disability (over 6 years of disease) and remission could be reached after 10–15 years from the onset in some cases [11].

NSSI and FEDs often coexist during adolescence with reciprocal influences on the severity of the clinical picture. Recent studies estimated a prevalence of 29–43% of NSSI in patients with BN and 14–21% in those with AN [13,14], although higher rates are reported for hospitalized patients up to 87.5% for BN and 30% for AN [15]. Importantly, all studies agree on the higher prevalence of NSSI in BN, as well as in the Binging/Purging subtype of AN, than in the Restricting subtype of AN, likely associated to the impulsive trait which is typical of those presentations characterized by NSSI [16]. Thus, it is reasonable to assume that a common etiopathology is shared between the two conditions.

Researchers hypothesized an integrated model based on the evidence of a low self-esteem and a low body acceptance in both conditions which have been related to dissociative symptoms and different forms of self-mutilation [17]. Several risk factors have been taken into account for the development of these associations, including individual and sociocultural, distal and proximal factors (e.g., early traumatic experiences, emotional dysregulation, cognitive distortions, social pressure). Sexual objectification of the female—and more recently male [18]—body also seems to drive the development of an unrealistic stereotypical “ideal body”.

Interestingly, a recent study [19] conducted on 73 female adolescent patients with AN found that 32 of them, who also had NSSI behaviors, displayed a greater prevalence of binging/purging behaviors, higher levels of both internalizing and externalizing symptoms, a lower cognitive functioning, and lower scores in a measure of self-directedness and cooperativeness. Particularly, their cognitive profile, assessed by means of a standardized multicomponent scale, provided evidence for lower metacognitive/executive abilities in the AN + NSSI group, specifically associated to NSSI behaviors, despite a global intelligence quotient in the normal range.

## 2. Materials and Methods

### 2.1. Participants

We conducted a cross-sectional observational study. Our sample is composed of forty-five female adolescent inpatients and outpatients aged 11 to 18 years old (mean age = 14.82 ± 1.46 years) consecutively recruited at our third-level research hospital for child and adolescent psychiatry and psychopharmacology from October 2022 to October 2023. Eligibility criteria were defined as follows: age range from 11 to 18 years old; diagnosis of Mood Disorder (MD) with NSSI according to the DSM-5-TR-based research provisional criteria and/or diagnosis of Feeding and Eating Disorder (FED); absence of current psychotic symptoms; Full-Scale Intelligence Quotient (FSIQ) and/or General Ability Index (GAI) equal to or greater than 85. Informed consent was obtained from all subjects involved in the study and their parents. The study was conducted in accordance with the Declaration of Helsinki, and approved by the Institutional Review Board of the Meyer Childrens’ Hospital (Regional Pediatric Ethics Committee of Tuscany, code Affect2022, approval date 20 September 2022).

Patients underwent a full diagnostic assessment including parent- and self-rated clinical questionnaires and a DSM-5-based standardized semi-structured interview—the Kiddie Schedule for Affective Disorders and Schizophrenia—present and lifetime version (K-SADS-PL) [20]—administered by trained child psychiatrists, as well as a neuropsychological evaluation based on the Wechsler Intelligence Scale for Children—fourth edition (WISC-IV) [21] or the Wechsler Adult Intelligence Scale—fourth edition (WAIS-IV) [22].

### 2.2. Self-Rated Questionnaires

The following self-rated questionnaires were administered to patients:

- the Youth Self Report—for youths aged 11 to 18 years (YSR—11/18) [23], a widely used, reliable, and validated child-report measure that assesses problem behaviors along three broadband scales (Internalizing, Externalizing, and Total Problems) and several empirically based syndromes and DSM-oriented scales;
- the Reactivity, Intensity, Polarity, and Stability—youth version (RIPoSt-Y) [24,25], a 31-item questionnaire aimed to assess the presence of ED along three main dimensions (Affective Instability, Emotional Reactivity, and Interpersonal Sensitivity) with corresponding cutoff scores and good validity and reliability;
- the Cyclothymic—Hypersensitive Temperament (CHT) [26,27] with 22 items to identify cyclothymic temperamental traits in young people aged 10 or older;
- the Multidimensional Anxiety Scale for Children—second edition (MASC-2) [28] and the Children Depression Inventory—second edition (CDI-2) [29], two clinical measures that, respectively, evaluate the presence of anxiety- and depression-related symptoms from 8 years of age and that have been validated elsewhere;
- the Deliberate Self-Harm Inventory (DSHI) [30], assessing frequency, duration, type, and severity of NSSI behaviors, and the Columbia Suicide Severity Rating Scale (C-SSRS) [31] as a validated and reliable screening tool for the identification of suicidal ideation and behaviors widely used in clinical and nonclinical settings;
- the Barratt Impulsiveness Scale (BIS-11) [32], a questionnaire aimed to provide a clinical measure of impulsivity along six first-order dimensions (Attention, Motor, Self-Control, Cognitive Complexity, Perseverance, and Cognitive Instability) and three second-order factors (Attentional, Motor, Non-Planning Impulsiveness) with good validity and reliability;
- the Personality Inventory for DSM-5—brief form (PID-5-BF) [33] which evaluates the personological profile of participants based on five domains (Negative Affectivity, Detachment, Antagonism, Disinhibition, and Psychoticism) displaying good validity and reliability;
- the Center for Substance Abuse Prevention—youth survey (CSAP) [34], a validated screening tool with its 26 items for the assessment of the use of alcohol, tobacco, and other substances;
- the Eating Disorder Inventory—third edition (EDI-3) [35] and the Body Uneasiness Test (BUT) [36] specifically assessing eating disorders-related symptoms and satisfaction about body image, respectively, with good validity and reliability.

All the self-rated questionnaires used in this study have been validated in the population in developmental age.

### 2.3. Parent- and Clinician-Rated Measures

The following self-rated questionnaires were administered to caregivers: the Child Behavior Checklist for youths ages 6 to 18 years (CBCL—6/18) [23], a widely used parent-report measure that assesses problem behaviors along three broadband scales (Internalizing, Externalizing, and Total Problems) and several empirically based syndromes and DSM-oriented scales; and the Behavior Rating Inventory of Executive Function (EF)—second edition (BRIEF2) [37] which provides a clinical measure of executive functioning in daily life through nine subscales along three major dimensions (Emotional, Behavioral, and Cognitive Regulation Indexes) and one General Executive Composite.

Finally, clinicians rated the Clinical Global Impression Severity Scale (CGI-S) [38], providing a severity score of the clinical picture on a Likert scale from 1 to 7, and the Children’s Global Assessment Scale (CGAS) [39], a rating scale assessing functional impairment of patients. They also reported relevant clinical information including number of previous attempted suicides, type of pharmacological and non-pharmacological interventions, weight, height, and body mass index (BMI).

### 2.4. Statistics

Statistical analyses were performed with RStudio^®^ software (version R 4.0.2). An Analysis of Variance (ANOVA) was conducted to assess statistically significant effects of the clinical groups on variables with continuous distribution. The χ^2^ test was instead applied to verify statistically significant differences in the distribution of nominal variables among groups. Finally, a Principal Component Analysis (PCA) was conducted to reduce the dimensionality of our large dataset. A preliminary Parallel Analysis was performed to define the number of components to extract among the 78 variables of interest. A PCA with Promax oblique rotation (n = 1000 iterations) was thus conducted on a subset of 32 patients with full observations according to the number of components to be extracted. Then, loadings on each component were computed and those variables with loadings equal to or greater than 0.6 were retained. Sums of z-scores of the variables for each component were estimated and compared among the three clinical groups by means of an ANOVA.

## 3. Results

Clinical and demographic variables with continuous and nominal distribution were initially compared among the three groups (NSSI, FED, and NSSI + FED). While age did not differ among groups, FED and NSSI + FED presented with a significantly lower BMI than NSSI (*p* < 0.001) (Table 1). The χ^2^ test revealed a greater rate of binging/purging behaviors specifically in the NSSI + FED group (*p* < 0.001), and a greater rate of food intake restrictions in both FED and NSSI + FED groups (*p* < 0.001) (Table 2). Among psychiatric comorbidities, Cyclothymic Disorder was significantly more prevalent in the NSSI and the NSSI + FED groups than the FED group, whereas Panic Disorder was more prevalent in the NSSI group than the FED group. CGAS scores were significantly lower in NSSI and NSSI + FED than FED alone (*p* = 0.007); CGI-S scores were instead higher in NSSI + FED than FED (*p* = 0.032) (Table 1). NSSI + FED patients were more frequently prescribed with SSRI as compared to the other two groups (*p* < 0.001), while NSSI patients were more frequently prescribed with Lithium Salts (*p* = 0.018) (Table 3). A non-significant difference emerged in the non-pharmacological interventions, with FED and NSSI + FED patients undergoing individual and family psychotherapy more frequently than NSSI (Table 3).

Significant differences also emerged in the clinical measures based on both self and parent reports, with NSSI + FED patients typically scoring more similarly to FED than NSSI, except for EF-related symptoms (Table S1). Specifically, the NSSI + FED group exhibited similar scores in the YSR questionnaire subscales as compared to the FED-only group, except for the Social Problems subscale (*p* = 0.0013), as well as similar scores in the CHT and two subscales of the PID (Antagonism and Psychoticism) (Table S1). Finally, similar scores were also found between the NSSI and the NSSI + FED groups in the Sustained Attention subscale of the BIS questionnaire (Table S1). The χ^2^ test also revealed similar rates of Suicidal Ideation in NSSI and NSSI + FED which is significantly greater than in FED, both structured (*p* = 0.024) and non-structured (*p* = 0.0122) (Table 4). No significant difference was identified in substance use habits (Table 4).

The Parallel Analysis revealed that four principal components could be extracted from the 78 variables of interest. The four components extracted by means of the PCA were labeled as follows: “Thought Problems”, “Body Image”, “Anxiety”, and “Metacognition”; specifically, this latter was defined as the cognitive process aimed to understand how to maximize learning and decision-making. The four identified principal components included the following variables:

- Thought Problems: YSR-11/18 Thought Problems, EDI-3 Interpersonal Alienation, EDI-3 Perfectionism, PID-5-BF Psychoticism, EDI-3 Obsessive Control, YSR-11/18 Somatic Complaints, EDI-3 Interpersonal Problems Composite, EDI-3 Affective Problems Composite, BIS-11 Cognitive Instability, PID-5 Antagonism, EDI-3 Interoceptive Deficits, EDI-3 Emotional Dysregulation, EDI-3 Interpersonal Insecurity, RIPoSt-Y Affective Instability, PID-5 Negative Affectivity, CDI-2 Negative Mood, YSR-11/18 Aggressive Behavior, YSR-11/18 Withdrawn/Depressed;
- Body Image: EDI-3 Body Dissatisfaction, BUT Weight Phobia, BUT Body Image Worry, EDI-3 Eating Disorder Risk Composite, EDI-3 Drive for Thinness, BUT Compulsive Monitoring, BUT Depersonalization, EDI-3 Inconclusiveness, EDI-3 Low Self Esteem, BUT Avoiding, EDI-3 Personal Alienation;
- Anxiety: all MASC-2 subscales;
- Metacognition: BRIEF-2 Task Monitor, BRIEF-2 Plan/Organize, BIS-11 Motor Impulsiveness, BIS-11 Motor Instability, BRIEF-2 Inhibit, BRIEF-2 Organization of Materials, BRIEF-2 Initiate, BIS-11 Self Control, RIPoSt-Y Interpersonal Sensitivity, YSR-11/18 Attention Problems, BRIEF-2 Working Memory, BIS-11 Non-Planning Impulsivity.

No significant difference emerged in the “Thought Problems” and “Anxiety” components among the three groups, although NSSI patients exhibited almost-significantly higher scores than the NSSI + FED followed by the FED (Table 5). Significantly higher scores were found for the “Body Image” component in the NSSI + FED than the other two groups (*p* = 0.0005) (Table 5). As for the “Metacognition” component, a significant difference emerged among the three groups, with NSSI patients scoring significantly higher than FED (*p* = 0.0370), while NSSI + FED did not differ as compared to the other groups (Table 5).

## 4. Discussion

The present study aimed to compare clinical, psychopathological, and neuropsychological features among three groups of young patients with Mood Disorders with NSSI and/or Feeding and Eating Disorders consecutively recruited at our research hospital. We first found a significantly lower BMI detected in the FED and NSSI + FED groups than the NSSI-only group, while no difference emerged between the former two groups; this latter aspect further strengthens the need to monitor weight loss in these populations to prevent potentially fatal outcomes [40]; moreover, these data highlight the importance of closely monitoring weight in patients with self-harm.

In line with previous studies [16,41], a greater prevalence of binging/purging behaviors has been detected in the NSSI + FED group. Interestingly, this piece of evidence is also in line with a theoretical model proposed by Maestro et al. [40] based on Brooks et al. [42] suggesting a spectrum model of different forms of FED from more “restricting” to more “impulsive” phenotypes with potential underlying neurofunctional and genetic correlates. Specifically, the functional reduction—or at least dysregulation—of striatal dopaminergic circuits, combined with varying degrees of PFC-related cognitive control, contributes to the differential pathologies observed in AN, BN, and BED [43]. In addition, genetic data suggest potential polymorphisms for FED in the genes encoding BDNF, COMT, and 5HT2A [44,45], the interactions between which may contribute to a spectrum of disordered eating. Genetic data may provide ground for neuroimaging findings, in that BDNF is linked to synaptic plasticity in the mesolimbic reward pathway, whereas COMT is involved in the breakdown and clearance of dopamine arriving at the PFC. Interactions between these two systems, rather than isolated polymorphisms at each gene, may contribute to FED phenotypes and the neural activation observed in neuroimaging studies.

In line with this, a strong association between binging/purging subtypes of FED and NSSI behaviors has been demonstrated [46]. A study compared clinical and ED characteristics and psychopathological traits in a sample of 253 female adolescents with AN with or without NSSI and suggested that adolescents with AN and NSSI show peculiar clinical features with higher prevalence of binging/purging-type AN and more severe psychopathological traits than adolescents with AN without NSSI [47].

Nonetheless, the presence of restricting patterns does not exclude the occurrence of NSSI behaviors [48]. In this regard, it would be important to provide a more specific assessment and classification in the context of non-suicidal self-harm, in order to distinguish the more impulsive forms, implemented on the basis of the urge, and those premeditated with the presence of structured planning (impulsive versus premeditated NSSI [49]).

The NSSI + FED group in our study exhibited a greater prevalence of Cyclothymic Disorder than the FED-only group. Cyclothymic Disorder is known to be characterized by emotional dysregulation (ED) [50], interpersonal sensitivity, and affective reactivity [51]; these characteristics can lead these patients to a longer duration and intensity of their emotional experiences, which are consequently experienced as a continuous source of suffering. Moreover, a study confirmed that FED behaviors have a function in regulating emotions, including feelings of shame, especially body shame [52]. Previous studies found that ED might be regarded as a common substrate between FED and NSSI [53,54] or even a potential pathogenetic mechanism contributing to the occurrence of NSSI and suicidal behaviors in patients with FED [55]. Indeed, our results also showed that higher levels of suicidal ideation are detected in the NSSI + FED group than the FED group, both for structured and non-structured ideation. Our previous longitudinal study [9] conducted on young patients with Mood Disorders also confirmed that persistent NSSI behaviors is associated with a greater risk of suicidal ideation. The notion that NSSI is a risk factor for suicidal ideation and behavior was already known; thus, our paper supports and extends previous findings [56] and our contribution was to provide novel evidence for the notion that persisting, but not remitting, NSSI is associated with higher suicidal behavior at follow-up.

Clinical severity assessment based on CGAS scale identifies, in both groups with NSSI behaviors, the presence of marked deficits in global functioning in almost all areas of daily life, including social functioning. Similarly, mean scores in the CGI-S scale for both groups with NSSI behaviors reveal meaningful severity of symptoms with high interference with daily life functioning.

The current literature on the pharmacological treatment of patients with FED is still controversial, also in light of the possible adverse effects linked to the clinical impairment of these patients. SSRI received the greatest attention and may represent the treatment of choice for AN and AN-related depressive or anxiety symptoms [57]. In our sample, there is a more frequent prescription of serotoninergic agents, particularly Sertraline, in NSSI + FED patients as compared to NSSI and FED. Conversely, we found a more frequent prescription of Lithium Salts in NSSI patients as compared to the other two groups. Previous studies suggested that, whenever comorbid Mood Disorders, including Cyclothymia, and NSSI behaviors, are present, the risk of (hypo)manic switch induced by SSRI is known to be much more frequent and associated with impulsive behaviors such as suicidal behaviors [58]. The data we collected therefore show that the target of pharmacological treatment related to NSSI + FED patients was mostly based on the presence of FED rather than on the presence of NSSI and comorbidities (Cyclothymia), while in patients with NSSI mood stabilizers were preferred.

As far as non-pharmacological treatments, it is important to highlight that given the clinical severity of the patients involved in the study, all of them were suggested to start individual psychotherapy. Regarding psychotherapeutic treatment for eating disorders characterized by binge eating and purging behaviors in adolescents, there are few randomized controlled trials. In view of the comorbidity of this type of conduct with ED and suicidal and self-injurious ideation, a study examined the effectiveness of an outpatient Dialectical Behavior Therapy (DBT) program for adolescents with symptoms of bulimia nervosa, suicide attempts, and non-suicidal self-harm [59]. Of the ten eligible participants enrolled, seven completed 6 months of treatment and 6 months of follow-up assessments. At post-treatment, participants had significantly reduced self-harm, frequency of objective binge eating episodes, frequency of eliminations, and Global Eating Disorder Examination assessment. At follow-up, six participants had no more self-injurious behaviors and three participants had no more binge eating.

Regarding a long-term perspective of the efficacy of DBT with adolescent subjects with self-harm and suicidal ideation, Mehlum et al. carried out a prospective follow-up of 3 years, noting that the effectiveness of DBT-A remained superior to that of traditional psychotherapy in reducing the frequency of self-harm, despite the fact that there were no statistically significant differences between the groups regarding suicidal ideation, depressive symptoms, and global level of functioning [60].

Finally, we performed a Principal Component Analysis on our dataset that allowed us to synthesize our findings along four main dimensions of functioning. As for “Body Image”, the NSSI + FED group exhibited the greatest impairment compared to the other groups. Negative feelings and attitudes toward one’s own body, self-image dissatisfaction, and disruption of body image have been previously linked to NSSI behaviors in both clinical and community samples [61]. A recent study by Perez and colleagues [62] examined patients with restricting and binging/purging subtypes of FED, with and without NSSI behaviors, showing a significantly greater impairment of self-image in those with NSSI. Particularly, patients with a recent or past history of NSSI showed a more impaired evaluation of their own body image and a greater dissatisfaction with different parts of their own body [62]. NSSI, especially if associated with FED, is characterized by negative feelings linked to body perception, physical contact, and self-protection [48,50].

An important role in the pathogenesis of self-harm also appears to be the presence of early life stress events including neglect and parental invalidation that prevent the development of positive self-esteem and self-image and the acquisition of appropriate emotional self-regulation strategies [58]. Interestingly, self-care and protection are not innate instincts, but they develop through imitation and interiorization of active parental care; on the other hand, neglect-related frustration and sorrow may evoke offensive attitudes toward the body [63]. In line with this, self-injury, food restriction, and binging/purging behaviors are often actively engaged with the explicit intent to cause damage to the body both in short and long term, sometimes with suicidal intention [64]. It is noteworthy that body dissatisfaction, self-care detachment, and disrupted self-protection are also linked to a reduced sensitivity to pain, which favors the implementation of self-injurious behaviors as a dysfunctional strategy of managing negative feelings [63,64]

As for “Metacognition”, we found significantly greater metacognitive impairment in the NSSI group than in the FED group; the metacognitive abilities of the FED + NSSI group are superior to those of the NSSI group but lower than those of the FED group. Previous studies [64] found that several metacognitive skills, based on clinical observation, are less frequently employed in highly compromised patients with FED. The studies also show that non-suicidal self-harm, BN, and BED appear to be related to aspects of impulsivity, lack of self-control, action inhibition deficits, and poor planning and decision making [62,63,64].

Limitations of our study include the low number of patients and the single-site recruitment design, which might prevent the generalizability of our findings. Future studies should aim to identify early risk markers of subsequent development of comorbid clinical pictures, as well as investigate premorbid signs of the aforementioned features in order to examine whether low self-esteem, disrupted self-image, and metacognitive deficits might predict NSSI and FED behaviors.

## 5. Conclusions

Our work carried out in a third-level research hospital for child and adolescent psychiatry and psychopharmacology from all over the nation allowed us to cross-search various departments of the structure by collecting a homogeneous sample that was at the same time varied.

In line with the evidence provided by the previous literature on the topic, our study confirmed that patients with comorbid NSSI and FED exhibit distinct clinical features as compared to non-comorbid conditions. Such patients are indeed characterized by the strongest negative attitudes and feelings toward their own body, limited metacognitive skills with high impulsivity, and a marked global impairment of functioning.

The main contribution of the present study is therefore to support and suggest the identification of specific assessment strategies and follow-up monitoring as well as dedicated evidence-based intervention protocols for this subtype of patient intended to identify the most appropriate pharmacological and psychotherapeutic treatment approaches.

## Figures and Tables

**Table 1:** Demographic and clinical features of patients.

	FED	NSSI + FED	NSSI	F	*p*	Post-Hoc
Age (years)	14.4 ± 1.682	14.6 ± 1.242	15.267 ± 1.28	1.542	0.226	
Weight (kg)	39.557 ± 5.084	44.463 ± 8.202	61.273 ± 16.709	15.677	<0.001	NSSI > FED NSSI > NSSI + FED
Height (cm)	158.267 ± 4.395	159.6 ± 6.47	160.7 ± 6.488	0.647	0.529	
BMI (kg/cm^2^)	15.799 ± 1.969	17.387 ± 2.704	23.605 ± 5.641	17.807	<0.001 *	NSSI > FED NSSI > NSSI + FED
CGI-S score	4.867 ± 0.640	5.467 ± 0.640	5.333 ± 0.617	3.722	0.032 *	NSSI + FED > FED
C-GAS scores	42.333 ± 11.159	30.333 ± 13.689	29.4 ± 10.473	5.553	0.007 *	FED > NSSI + FED FED > NSSI
WISC-IV—VCI	114.533 ± 12.455	104.571 ± 10.624	113.067 ± 18.695	1.992	0.149	
WISC-IV—VSI	116.733 ± 16.064	112.867 ± 12.426	114 ± 12.75	0.309	0.736	
WISC-IV—WMI	101.571 ± 12.978	94.5 ± 12.757	94.667 ± 24.743	0.716	0.495	
WISC-IV—PSI	99.667 ± 10.635	98.933 ± 13.781	100.533 ± 18.007	0.046	0.955	
WISC-IV—FSIQ	112.467 ± 13.25	105.714 ± 12.175	111.769 ± 25.962	0.608	0.549	

* *p* < 0.05

**Table 2:** Clinical diagnoses and comorbidities of patients.

	FED	NSSI + FED	NSSI	χ^2^	*p*
Anorexia Nervosa—restricting	14 (93.33%)	12 (80%)	1 (6.67%)	27.222	<0.001 *
Anorexia Nervosa—binge/purging	0 (0.00%)	7 (46.67%)	0 (0.00%)	16.579	<0.001 *
Bulimia Nervosa	1 (6.67%)	0 (0.00%)	0 (0.00%)	2.045	0.360
Avoidant/Restrictive Food Intake	1 (6.67%)	0 (0.00%)	0 (0.00%)	2.045	0.360
Bipolar Disorder—type 1	1 (6.67%)	2 (13.33%)	1 (6.67%)	0.549	0.760
Bipolar Disorder—type 2	2 (13.33%)	6 (40%)	2 (13.33%)	4.114	0.128
Cyclothymic Disorder	4 (26.67%)	10 (66.67%)	14 (93.33%)	14.370	<0.001 *
Major Depression Disorder	7 (46.67%)	7 (46.67%)	1 (6.67%)	7.200	0.027 *
Social Anxiety Disorder	5 (33.33%)	8 (53.33%)	9 (60%)	2.312	0.315
Generalized Anxiety Disorder	9 (60%)	11 (73.33%)	12 (80%)	1.514	0.469
Panic Disorder	1 (6.67%)	5 (33.33%)	11 (73.33%)	14.370	<0.001 *
Separation Anxiety Disorder	2 (13.33%)	1 (6.67%)	2 (13.33%)	0.450	0.799
Obsessive-Compulsive Disorder	4 (26.67%)	0 (0.00%)	3 (20%)	4.398	0.111
Post-Traumatic Stress Disorder	0 (0.00%)	2 (13.33%)	2 (13.33%)	2.195	0.334
Conduct Disorder	1 (6.67%)	0 (0.00%)	0 (0.00%)	2.045	0.360
Autism Spectrum Disorder	2 (13.33%)	2 (13.33%)	5 (33.33%)	2.500	0.287
Attention-Deficit/Hyperactivity Disorder	2 (13.33%)	2 (13.33%)	7 (46.67%)	6.016	0.049 *
Specific Learning Disability	0 (0.00%)	4 (26.67%)	3 (20%)	4.398	0.111
Personality Disorder—cluster B	6 (40%)	6 (40%)	11 (73.33%)	4.447	0.108

* *p* < 0.05

**Table 3:** Pharmacological treatments and psychotherapy interventions.

	FED	NSSI + FED	NSSI	χ^2^	*p*
Atypical Antipsychotics	4 (26.67%)	7 (46.67%)	5 (33.33%)	1.358	0.507
Risperidone	1 (6.67%)	1 (6.67%)	0 (0.00%)	1.047	0.593
Aripiprazole	2 (13.33%)	3 (20%)	3 (20%)	0.304	0.859
Lurasidone	0 (0.00%)	1 (6.67%)	0 (0.00%)	2.045	0.360
Quetiapine	2 (13.33%)	1 (6.67%)	3 (20%)	1.154	0.562
Olanzapine	0 (0.00%)	1 (6.67%)	0 (0.00%)	2.045	0.360
Methylphenidate	0 (0.00%)	1 (6.67%)	0 (0.00%)	2.045	0.360
Antidepressants	3 (20%)	10 (66.67%)	3 (20%)	9.504	0.009 *
Fluoxetine	0 (0.00%)	0 (0.00%)	1 (6.67%)	2.045	0.360
Sertraline	2 (13.33%)	11 (73.33%)	1 (6.67%)	18.871	<0.001 *
Fluvoxamine	1 (6.67%)	0 (0.00%)	0 (0.00%)	2.045	0.360
Citalopram	0 (0.00%)	0 (0.00%)	1 (6.67%)	2.045	0.360
Mood Stabilizers	1 (6.67%)	3 (20%)	7 (46.67%)	6.738	0.034 *
Carbamazepine	0 (0.00%)	2 (13.33%)	2 (13.33%)	2.195	0.334
Lithium Salts	0 (0.00%)	1 (6.67%)	5 (33.33%)	8.077	0.018 *
Valproic Acid	1 (6.67%)	0 (0.00%)	0 (0.00%)	2.045	0.360
Gabapentin	1 (6.67%)	0 (0.00%)	0 (0.00%)	2.045	0.360
Individual Psychotherapy	8 (53.33%)	10 (66.67%)	5 (33.33%)	3.379	0.185
Familiar Psychotherapy	3 (20%)	6 (40%)	1 (6.67%)	4.886	0.087
Dialectical Behavioral Therapy	1 (6.67%)	0 (0.00%)	1 (6.67%)	1.047	0.593

* *p* < 0.05.

**Table 4:** Clinical questionnaires—nominal variables.

	FED	NSSI + FED	NSSI	χ^2^	*p*
DSHI—Cutting	–	11 (73.33%)	14 (93.33%)	4.84	0.3295
DSHI—Cigarette Burning	–	2 (13.33%)	0 (0%)	0	0.4828
DSHI—Lighter Burning	–	2 (13.33%)	1 (6.67%)	0.48	1.0000
DSHI—Words Carving	–	1 (6.67%)	1 (6.67%)	1	1.0000
DSHI—Pictures Carving	–	6 (40%)	3 (20%)	0.39	0.4270
DSHI—Scratching	–	2 (13.33%)	1 (6.67%)	0.48	1.0000
DSHI—Biting	–	3 (20%)	1 (6.67%)	0.3	0.5977
DSHI—Acid Dripping	–	1 (6.67%)	0 (0%)	0	1.0000
DSHI—Rubbing	–	1 (6.67%)	1 (6.67%)	1	1.0000
DSHI—Scrubbing	–	1 (6.67%)	0 (0%)	0	1.0000
DSHI—Sharp Objects	–	2 (13.33%)	0 (0%)	0	0.4828
DSHI—Total Score	–	13 (86.67%)	15 (100%)	10	0.4828
CSSR-S—Ideation	4 (26.67%)	12 (80%)	9 (60%)	8.82	0.0122 *
CSSR-S—Non-Structured	4 (26.67%)	12 (80%)	9 (60%)	8.82	0.0122 *
CSSR-S—Structured	0 (0%)	5 (33.33%)	6 (40%)	7.46	0.0240 *
CSSR-S—Behavior	0 (0%)	3 (20%)	3 (20%)	3.46	0.1771
Previous Suicidal Attempt	0 (0%)	1 (6.67%)	3 (20%)	3.84	0.1465
CSAP—Cigarette Smoking	1 (6.67%)	5 (33.33%)	2 (13.33%)	3.95	0.1386
CSAP—Cannabis	0 (0%)	2 (13.33%)	0 (0%)	4.19	0.1233
CSAP—Alcohol	2 (13.33%)	6 (40%)	4 (26.67%)	2.73	0.2557

* *p* < 0.05.

**Table 5:** Principal Components Comparison.

	F	*p*	Post-Hoc
Thought Problems	2.70	0.0810	
Body Image	9.12	0.0005 ***	NSSI + FED > FED; NSSI + FED > NSSI
Anxiety	0.13	0.8700	
Metacognition	3.61	0.0370 *	FED > NSSI

* *p* < 0.05; *** *p* < 0.001.

## Data Availability

Dataset available on request from the authors.

## Supplementary Materials

The following supporting information can be downloaded at: https://www.mdpi.com/article/10.3390/children11080947/s1, Table S1: Clinical questionnaires—continuous variables.
