# Supplementary material for: Non-Suicidal Self-Injury in Eating and Feeding Disorder Patients: Characteristics and Clinical Implications in a Group of Referred Female Adolescents
Source: Children (Basel). 2024 Aug 6;11(8):947. doi: 10.3390/children11080947 (PMC11353179; doi:10.3390/children11080947)
Supplement: Supplementary file 1 [file children-11-00947-s001.zip › children-3099734-supplementary.pdf]

Supplemental Materials

Table S1. Clinical questionnaires – continuous variables

	F	p	Post-Hoc
YSR – Anxious/Depressed	1.8205	0.1748	
YSR – Withdrawn/Depressed	2.4427	0.0995	
YSR – Somatic Complaints	7.86	0.0013*	NSSI > FED; NSSI > NSSI+FED
YSR – Social Problems	5.8877	0.0057*	NSSI+FED > FED; NSSI > FED
YSR – Thought Problems	9.9702	0.0003***	NSSI > FED; NSSI > NSSI+FED
YSR – Attention Problems	11.7026	0.0001***	NSSI > FED; NSSI > NSSI+FED
YSR – Rule Breaking Behaviors	10.9894	0.0002***	NSSI > FED; NSSI > NSSI+FED
YSR – Aggressive Behaviors	5.0016	0.0114*	NSSI > FED
YSR – Internalizing Problems	3.7798	0.0311*	NSSI > FED
YSR – Externalizing Problems	6.5935	0.0033*	NSSI > FED; NSSI > NSSI+FED
YSR – Total Problems	10.3809	0.0002***	NSSI > FED; NSSI > NSSI+FED
CBCL – Anxious/Depressed	0.9449	0.3968	
CBCL – Withdrawn/Depressed	1.4519	0.2456	
CBCL – Somatic Complaints	3.3425	0.045*	NSSI > FED
CBCL – Social Problems	4.8556	0.0127*	NSSI > FED
CBCL – Thought Problems	4.1316	0.023*	NSSI+FED > FED; NSSI > FED
CBCL – Attention Problems	2.0522	0.1411	
CBCL – Rule Breaking Behaviors	0.696	0.5043	
CBCL – Aggressive Behaviors	4.2926	0.0201*	NSSI > FED
CBCL – Internalizing Problems	1.8821	0.1649	
CBCL – Externalizing Problems	1.8662	0.1673	
CBCL – Total Problems	2.0847	0.137	
CBCL – Anxious/Depressed	3.4235	0.0422*	NSSI > FED
MASC – Specific Phobias	0.1489	0.8621	
MASC – Generalized Anxiety	0.2245	0.7999	
MASC – Social Anxiety	1.313	0.2804	
MASC – Humiliation/Reject	1.6554	0.2038	
MASC – Performance Fear	0.7167	0.4945	
MASC – Obsessive/Compulsive	0.0903	0.9138	
MASC – Physical Symptoms	0.9031	0.4134	
MASC – Panic	0.788	0.4617	
MASC – Tense/Restless	0.2512	0.7791	
MASC – Harm Avoidance	0.6042	0.5514	
MASC – Total Score	0.3484	0.708	
CDI – Total Score	4.6138	0.0156*	NSSI > FED
CDI – Emotional Problems	1.9914	0.1495	
CDI – Negative Mood	0.5212	0.5977	
CDI – Negative Self-Esteem	2.1662	0.1276	
CDI – Functional Problems	2.4179	0.1017	
CDI – Ineffectiveness	2.5932	0.087	
CDI – Interpersonal Problems	0.3472	0.7087	
CHT – Total Score	4.1281	0.0233*	NSSI > FED; NSSI > NSSI+FED
RIPoSt-Y – Affective Instability	6.4384	0.0037*	NSSI > FED

RIPoSt-Y – Emotional Reactivity	4.8574	0.0128*	NSSI > FED
RIPoSt-Y – Interpersonal Sensitivity	1.2139	0.3075	
EDI – Drive for Thinness	6.0024	0.0052*	NSSI < NSSI+FED
EDI – Bulimia	1.9958	0.1489	
EDI – Body Dissatisfaction	5.9221	0.0055*	NSSI < NSSI+FED
EDI – Low Self Esteem	2.5471	0.0906	
EDI – Personal Alienation	3.1811	0.052	
EDI – Interpersonal Insecurity	1.3059	0.282	
EDI – Interpersonal Alienation	4.5353	0.0166*	NSSI > FED
EDI – Interoceptive Deficits	1.3798	0.2631	
EDI – Emotional Dysregulation	2.9656	0.0627	
EDI – Perfectionism	0.6061	0.5503	
EDI – Ascetism	1.3462	0.2715	
EDI – Maturity Fear	0.2124	0.8095	
EDI – Eating Disorder Risk Composite	3.2386	0.0494*	NSSI < NSSI+FED
EDI – Inconclusiveness	2.5621	0.0894	
EDI – Interpersonal Problems	2.2667	0.1165	
EDI – Affective Problems	2.899	0.0664	
EDI – Obsessive Control	1.152	0.326	
EDI – General Index	2.1563	0.1287	
BUT – Global Severity Index	9.1784	0.0005***	NSSI < FED; NSSI < NSSI+FED
BUT – Weight Phobia	8.4434	0.0008*	NSSI < FED; NSSI < NSSI+FED
BUT – Body Image Worry	6.4999	0.0035*	NSSI < NSSI+FED
BUT – Avoiding	5.5439	0.0073*	NSSI < NSSI+FED
BUT – Compulsive Monitoring	9.4487	0.0004***	NSSI < NSSI+FED
BUT – Depersonalization	4.1476	0.0227*	NSSI < NSSI+FED
PID-5-BF – Negative Affectivity	1.3195	0.2789	
PID-5-BF – Detachment	2.1092	0.135	
PID-5-BF – Antagonism	4.9314	0.0123*	NSSI > FED; NSSI > NSSI+FED
PID-5-BF – Disinhibition	8.464	0.0009*	NSSI > FED; NSSI > NSSI+FED
PID-5-BF – Psychoticism	5.3272	0.0091*	NSSI > FED
BIS-11 – Attention	7.9713	0.0012*	NSSI+FED > FED; NSSI > FED
BIS-11 – Cognitive Instability	2.7943	0.0731	
BIS-11 – Motor Instability	4.5162	0.017*	NSSI > FED
BIS-11 – Perseverance	3.8466	0.0297*	NSSI > FED
BIS-11 – Self-Control	0.0754	0.9275	
BIS-11 – Cognitive Complexity	1.7414	0.1883	
BIS-11 – Attentional Impulsivity	7.8465	0.0013*	NSSI > FED
BIS-11 – Motor Impulsivity	6.8667	0.0027*	NSSI > FED; NSSI > NSSI+FED
BIS-11 – Non-Planning Impulsivity	0.8436	0.4377	
BIS-11 – Total Score	6.7064	0.0031*	NSSI > FED
BRIEF-2 – Inhibit	2.6415	0.084	
BRIEF-2 – Self Monitor	1.9961	0.1495	
BRIEF-2 – Shift	0.4316	0.6526	
BRIEF-2 – Emotional Control	0.3302	0.7208	
BRIEF-2 – Initiate	2.0698	0.1399	
BRIEF-2 – Working Memory	5.0022	0.0116*	NSSI > FED
BRIEF-2 – Plan/Organize	3.432	0.0424*	NSSI > FED
BRIEF-2 – Task Monitor	6.1828	0.0047*	NSSI > FED; NSSI > NSSI+FED

BRIEF-2 – Organization of Materials	4.7868	0.0138*	NSSI > FED
BRIEF-2 – Behavioral Regulation Index	5.4453	0.0082*	NSSI > FED
BRIEF-2 – Emotional Regulation Index	2.3621	0.1076	
BRIEF-2 – Cognitive Regulation Index	5.5389	0.0076*	NSSI > FED
BRIEF-2 – General Executive Composite	3.3383	0.0459*	NSSI > FED

* $p < 0.05$, *** $p < 0.001$.
